# Safe and Effective Balloon Pulmonary Angioplasty in the Outpatient Setting: The Michigan Medicine Experience

**DOI:** 10.1016/j.jscai.2023.100589

**Published:** 2023-02-02

**Authors:** Lucas Rich, Nimai Patel, Syed N. Hyder, Hitinder Gurm, Victor Moles, Prachi P. Agarwal, Scott Visovatti, Jonathan Haft, Thomas Cascino, Vallerie V. Mclaughlin, Vikas Aggarwal

**Affiliations:** a Department of Internal Medicine, University of Michigan, Ann Arbor, Michigan; b Division of Cardiology (Frankel Cardiovascular Center), Department of Internal Medicine, University of Michigan, Ann Arbor, Michigan; c Division of Cardiothoracic Radiology, Department of Radiology, University of Michigan, Ann Arbor, Michigan; d Division of Cardiovascular Medicine, Department of Internal Medicine, The Ohio State University, Columbus, Ohio; e Department of Cardiac Surgery, University of Michigan, Ann Arbor, Michigan; f Section of Cardiology, Department of Internal Medicine, Veterans Affairs Medical Center, Ann Arbor, Michigan

**Keywords:** balloon pulmonary angioplasty, chronic thromboembolic pulmonary disease, chronic thromboembolic pulmonary hypertension, outpatient interventional procedures

## Abstract

**Background:**

Balloon pulmonary angioplasty (BPA) is currently performed at select centers worldwide, with the current standard of practice being postprocedural inpatient monitoring for 24 to 72 hours. We sought to evaluate the safety and efficacy of BPA in a cohort of patients with chronic thrombo-embolic pulmonary disease (CTEPD) and chronic thromboembolic pulmonary hypertension (CTEPH) and outline a protocol for implementation in the outpatient setting.

**Methods:**

All patients with distal, inoperable CTEPH, residual symptoms after pulmonary endarterectomy, or symptomatic CTEPD from July 1, 2020, to June 30, 2022, were evaluated by a multidisciplinary chronic thromboembolic pulmonary hypertension team for consideration of BPA. Patients undergoing each BPA session adhered to a regimented protocol developed and implemented at our institution. Safety and efficacy were retrospectively evaluated with a mean follow-up time of 8.5 months.

**Results:**

Eighteen patients underwent a total of 78 BPA sessions. Overall, there was a significant improvement in World Health Organization functional class and mean improvement in 6-minute walking distance of +67 m. Hemodynamic parameters significantly improved with a mean decrease in mean pulmonary artery pressure and pulmonary vascular resistance of 7.3 ± 5.8 mm Hg and 1.7 ± 1.5 Wood units, respectively (*P* <.05). Complication rates were low with 3 (3.9%) of 78 patients developing scant hemoptysis and 1 (1.3%) of 78 experiencing vascular injury requiring inpatient hospitalization.

**Conclusions:**

BPA is both safe and effective when implemented in the outpatient setting using a regimented protocol provided there are necessary contingencies in place.

## Highlights

• Balloon pulmonary angioplasty is currently performed at expert centers worldwide in patients with chronic thromboembolic pulmonary hypertension and symptomatic chronic thromboembolic pulmonary disease.
• Balloon pulmonary angioplasty has been shown to improve both symptoms and hemodynamic parameters.
• However, balloon pulmonary angioplasty is not without risk, and potential complications include vascular injury and pulmonary edema.
• The current standard of practice is inpatient postprocedural monitoring for 24 to 72 hours.
• We present an outpatient balloon pulmonary angioplasty protocol that results in efficacy comparable with recent multicenter registries and a low complication rate requiring inpatient hospitalization.

## Central Illustration

**Figure:**
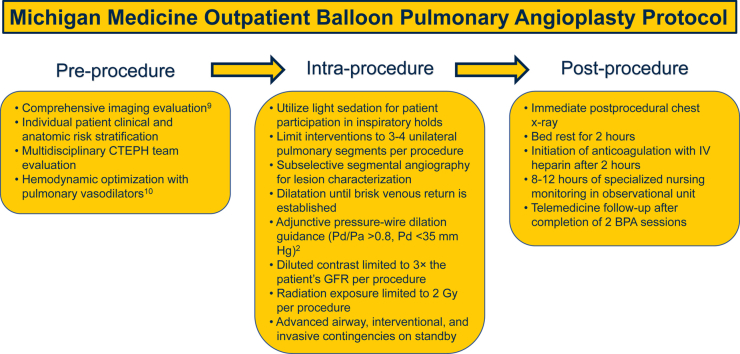

The role of balloon pulmonary angioplasty (BPA) in inoperable and residual chronic thromboembolic pulmonary hypertension (CTEPH) is well established, and evidence for the efficacy of BPA in select, symptomatic patients with chronic thromboembolic pulmonary disease (CTEPD) without pulmonary hypertension continues to evolve.^1^, ^2^, ^3^

Although the use of BPA continues to grow worldwide, it is important to note that it continues to be a high-risk percutaneous procedure with significant rates of complications, including vascular injury and pulmonary edema.^4^ BPA is currently performed at expert CTEPH centers worldwide, and the current practice at most centers is to observe these patients for 24 to 72 hours after each BPA session, given the risk of postprocedural complications.^2^^,^^5^^,^^6^

The COVID-19 pandemic introduced many challenges to the care of these patients, including the limited availability of intensive care and step-down hospital beds and a small but real risk of COVID-19 transmission in the periprocedural setting. Given the ongoing stress on health care resources, there is keen interest in determining how best to safely deliver interventional procedures to patients in the outpatient setting.^7^^,^^8^. We modified our pre-, intra-, and postprocedural protocols to safely perform BPA completely in the outpatient setting (Central Illustration). In this article, we present our protocol and our single-center experience detailing the safety and efficacy of BPA in an outpatient setting.

**Central Illustration:**
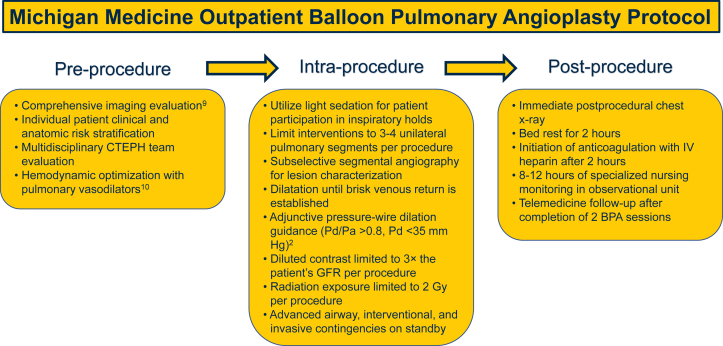
Michigan Medicine outpatient balloon pulmonary angioplasty protocol. BPA, balloon pulmonary angioplasty; CTEPH, chronic thromboembolic pulmonary hypertension; GFR, glomerular filtration rate; IV, intravenous.

## Methods

### Patient selection and preprocedural work-up

From July 1, 2020, to June 30, 2022, patients with distal, inoperable CTEPH, residual symptoms after pulmonary endarterectomy, or symptomatic CTEPD were considered for BPA. Each patient underwent a comprehensive evaluation, including transthoracic echocardiography, ventilation/perfusion scanning, dual-energy computed tomography, and right heart catheterization with pulmonary angiography.^9^^,^^10^ An individualized risk profile was created for each patient based on clinical and anatomic disease burden. Table 1 highlights generally accepted clinical and anatomical factors that are considered high risk. Patients were discussed in a multidisciplinary CTEPH team meeting, including a pulmonary hypertension specialist, experienced cardiothoracic surgeon, interventional cardiologist, and radiologist experienced in pulmonary vascular imaging. Patients with moderate-to-severe pulmonary hypertension at baseline were first pretreated with maximally tolerated pulmonary vasodilator therapy at the discretion of the pulmonary hypertension specialist.^11^

**Table 1:** High-risk clinical and anatomic features.

Clinical:
• World Health Organization class 4 symptoms
• Physical exam findings or hemodynamics consistent with volume overload
• Severe pulmonary hypertension
• Significant underlying parenchymal lung disease
• Advanced age
• Underlying hemoglobinopathies and inflammatory disorders
• Residual CTEPH after pulmonary endarterectomy
• Advanced chronic kidney disease
Anatomic:
• Chronic total pouch occlusions
• Severely tortuous vessels with diffuse disease

CTEPH, chronic thromboembolic pulmonary hypertension.

### Intraprocedural considerations

Depending on disease burden, we typically aimed to perform complete revascularization in 2 to 6 outpatient procedural sessions.

Our general approach is to revascularize 3 to 4 segments per lung in each session. This is often driven by contrast dose limitations individualized to each patient’s baseline kidney function. Our protocol is to not exceed a contrast dose 3 times the glomerular filtration rate per session, and the radiation dose is limited to 2 Gy.^12^ Only 1 lung is treated per session because this approach allows for selective intubation and ventilation of the unintervened-upon lung in the event of a pulmonary artery branch perforation and hemorrhage.

We utilize workhorse coronary guidewires with a nonhydrophilic coated tip supported using a microcatheter. Deep inspiratory hold is used in most cases to minimize vessel foreshortening during lesion crossing. A telescoping guide extension catheter is sometimes used for subselective branch cannulation.

Subselective segmental angiography, in 2 orthogonal projections, is performed for target lesion identification and characterization, facilitating real-time risk: benefit analysis before intervention.^9^^,^^13^ Digital subtraction angiography is not used to minimize radiation exposure. Pressure guided lesion assessment, with a cut-off of Pd/Pa <0.8 (Pd: distal pressure and Pa: proximal pressure), is used to identify lesions with significant obstruction.^14^^,^^15^ Generally, balloons utilized in BPA are angiographically undersized to minimize the incidence of vascular injury. We believe that the gold standard marker for adequate dilation is reestablishment of brisk venous return.

### Monitoring and management of complications

#### During the procedure

The entirety of the procedure is performed utilizing light anesthesia to allow the patient to participate in deep breath holds. Inspiratory breath holds are recommended for optimal visualization of vessels in the middle and lower lobes.^9^ The patient is continuously monitored using a pulse oximeter for a decrease in oxygen saturation or for a subjective need for increased oxygen; if requirements escalate beyond 6 L/min, the procedure is aborted, and the patient may be placed on a continuous positive airway pressure machine. If hemoptysis occurs, the involved vessel is treated using balloon tamponade and immediate reversal of anticoagulation; in these instances, the procedure is aborted. Escalation of care may include advanced interventional techniques, selective intubation, and inpatient hospital admission.

#### Postprocedural

Immediately after the procedure, a baseline chest x-ray is obtained to rule out early reperfusion pulmonary edema and to serve as a comparison for subsequent evaluation of delayed reperfusion edema over the next 8 to 12 hours.^9^ The patient is placed on bed rest for 2 hours to allow for hemostasis of the vascular access site. Afterward, systemic anticoagulation with intravenous heparin infusion is reinitiated. In the event of hemoptysis during the procedure, anticoagulation is restarted 12 hours after procedure completion. The patient remains in a postprocedural monitoring unit for 8 to 23 hours. Every patient is monitored overnight and discharged in the morning. These observation beds in our outpatient cardiac procedures recovery unit are not licensed in-hospital beds (under the hospital’s certificate of need) but are staffed with trained nurses 24 hours a day. Nursing staff caring for such patients undergo extensive additional education in identification of BPA complications, including vascular access site closure, evidence of lung injury, and reperfusion pulmonary edema. If needed, escalation of care via transfer to inpatient setting is always available. A telemedicine visit is scheduled after the completion of 2 sequential BPA sessions (1 week apart), to monitor for complications, assess symptomatic improvement, and reassess the need for future sessions.

### Statistical analysis

Continuous data were summarized with mean and SD and categorical data were summarized using count and percentage. Differences in mean parameters pre- and post-BPA were calculated using paired sample *t* testing. Two-tailed *P* value of <.05 was considered statistically significant.

## Results

### Patient characteristics

Eighteen patients (16 with CTEPH and 2 with symptomatic CTEPD without pulmonary hypertension) underwent a cumulative 78 BPA procedures at our center using this protocol. Patient demographics and indications for BPA are summarized in Table 2. The mean age was 61.7 years (range, 37-81 years). The population was predominately female (61%) and White (83%). There was a known clinical history of deep venous thrombosis and pulmonary embolism in 56% of patients. An underlying thrombophilia disorder was identified in 6 patients (33%).

**Table 2:** Patient demographics and indication for BPA.

Age, y	61.3 ± 15.8
Women	11 (61%)
Race	
White	15 (83%)
African American	2 (11%)
Pacific Islander	1 (6%)
Body mass index, kg/m^2^	27.9 ± 5.1
Prior deep venous thrombosis	10 (56%)
Prior pulmonary embolism	10 (56%)
Hypertension	8 (44%)
Hyperlipidemia	5 (28%)
Chronic obstructive lung disease	1 (6%)
Obstructive sleep apnea	7 (39%)
Chronic kidney disease	1 (6%)
Atrial fibrillation	3 (18%)
Thrombophilia	
Antiphospholipid syndrome	2 (11%)
Polycythemia	1 (6%)
Prothrombin gene mutation	1 (6%)
Hereditary spherocytosis	1 (6%)
Factor V Leiden	1 (6%)
Anticoagulation	
Warfarin	2 (12%)
Rivaroxaban	8 (44%)
Apixaban	8 (44%)
Indication for BPA	
Distal inoperable lesion	13 (72%)
Postpulmonary endarterectomy	4 (22%)
Patient preference	1 (6%)

Values are mean ± SD or n (%).

BPA, balloon pulmonary angioplasty.

**Figure 1:**
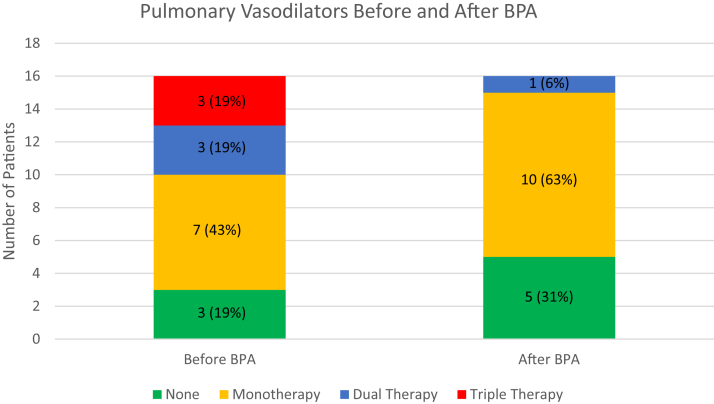
**Pulmonary vasodilator therapy in patients with chronic thromboembolic pulmonary hypertension before and after balloon pulmonary angioplasty.** BPA, balloon pulmonary angioplasty; CTEPH, chronic thromboembolic pulmonary hypertension.

Pulmonary vasodilator therapy before and after BPA is depicted in Figure 1. Prior to BPA, 13 out of 16 patients with CTEPH were on at least 1 pulmonary vasodilator with 11 (69%) of 16 patients being on riociguat. Only 22% of patients were on home oxygen therapy prior to BPA.

### BPA procedure

Characteristics of individual BPA procedures are summarized in Table 3. Each patient underwent a median of 5 BPA sessions (interquartile range, 3-8). Per procedure, the mean number of segments intervened upon was 2.3 ± 0.89 and the mean number of subsegmental branches was 3.7 ± 1.46. Table 4 summarizes our guide catheter utilization for subselective cannulation by each individual pulmonary segment.

**Table 3:** BPA procedural characteristics (per session).

Contrast dose, mL	166.2 ± 46.5
Contrast dose: glomerular filtration rate	2.1 ± 0.68
Radiation exposure, Gy	0.57 ± 0.51
Number of segments intervened	2.3 ± 0.89
Number of subsegmental branches intervened	3.7 ± 1.46
Fluoroscopy time, min	36.4 ± 12.8

Values are mean ± SD.

BPA, balloon angioplasty.

**Table 4:** Guide catheter utilization based on pulmonary segment.

Segmental branch	Guide catheter utilization (%)						
	JR4	Champ 1.5	Champ 2.5	MPA-2	EBU-3.5	EBU-4	Total Cannulations
Left apicoposterior trunk (A1-A2)	4 (80%)	–	–	–	–	1 (20%)	5
Left anterior segment (A3)	1 (25%)	–	2 (50%)	–	–	1 (25%)	4
Lingula (A4-A5)	19 (70%)	2 (7%)	5 (19%)	–	–	1 (4%)	27
Left superior segmental (A6)	6 (75%)	–	1 (12%)	–	1 (12%)	–	8
Left anterolateral trunk (A7-A9)	30 (86%)	2 (6%)	2 (6%)	1 (2%)	–	–	35
Left medial basal (A10)	10 (77%)	1 (8%)	2 (15%)	–	–	–	13
							
Right apical segment (A1)	5 (100%)	–	–	–	–	–	5
Right posterior segment (A2)	4 (100%)	–	–	–	–	–	4
Right anterior segment (A3)	19 (90%)	–	–	2 (10%)	–	–	21
Right middle lobe (A4-A5)	29 (97%)	–	1 (3%)	–	–	–	30
Right lower lobe anterolateral segmental trunk (A7-A8)	18 (90%)	–	1 (5%)	1 (5%)	–	–	20
Right lower lobe posteromedial segmental trunk (A9-A10)	29 (100%)	–	–	–	–	–	29

EBU, extra backup; JR, Judkins right; MPA, multipurpose A.

### BPA outcomes

**Figure 2:**
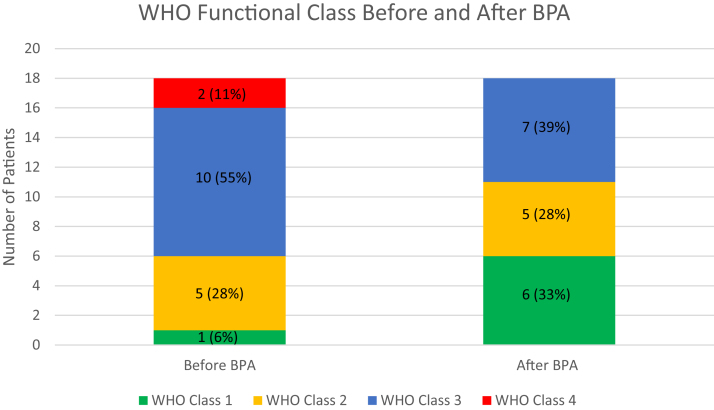
**World Health Organization functional class before and after balloon pulmonary angioplasty.** BPA, balloon pulmonary angioplasty; WHO, World Health Organization.

Patients’ functional and hemodynamic parameters are demonstrated in Table 5. At baseline, majority of patients were World Health Organization functional classes 3 and 4 as demonstrated in Figure 2. Baseline hemodynamic parameters indicated the presence of severe pulmonary hypertension was present prior to BPA.

**Table 5:** Functional and hemodynamic data before and after BPA.

	Before BPA	After BPA	*P*
6MWD, m	365.3 ± 146.3	432.6 ± 114.8	.09
RAP, mm Hg	13.4 ± 5.3	9.8 ± 2.5	.012
mPAP, mm Hg	43.4 ± 11.3	35.1 ± 10.2	<.01
Cardiac index, L/min/m^2^	2.6 ± 0.7	2.8 ± 5.0	.09
PVR, Wood units	5.1 ± 2.6	3.4 ± 1.4	<.01

Cardiac index reported via thermodilution,

6MWD, 6-minute walking distance; BPA, balloon pulmonary angioplasty; mPAP, mean pulmonary artery pressure; PVR, pulmonary vascular resistance; RAP, right atrial pressure.

After BPA, World Health Organization functional class and exercise tolerance improved. Furthermore, hemodynamic parameters, including mean pulmonary arterial pressure and pulmonary vascular resistance were significantly improved, with a mean improvement of 7.3 ± 5.8 mm Hg and 1.7 ± 1.5 Wood units, respectively. Mean follow-up time was 8.5 ± 5.9 months with sustained improvements in functional assessments.

### Complications

Complications occurred in 4 (5.1%) of 78 of procedures, with scant hemoptysis in 3 (3.8%) of 78 procedures and vascular perforation requiring coil embolization with inpatient admission in 1 (1.3%) of 78 procedures. There were no occurrences of reperfusion pulmonary edema or death within 30 days of the procedure.

## Discussion

Our experience highlights the successful implementation of a novel protocol that allows for safe and effective BPA in the outpatient setting. Our results demonstrate improvements in functional and hemodynamic parameters, as well as low complication rates, that are comparable with those reported based on multicenter registries.^5^^,^^6^^,^^16^, ^17^, ^18^ We believe that our low complication rates were the result of our use of an experienced, multidisciplinary BPA team with refined technical expertise.^19^^,^^20^ Thus, we do not recommend new programs initiating BPA implement our outpatient postprocedural monitoring. Instead, our outpatient approach should only be implemented once enough experience is gained using an inpatient postprocedural monitoring approach.

Critical to the overall success of our program is a preprocedural phase utilizing a comprehensive imaging evaluation as well as hemodynamic optimization with medical therapy. An earlier report published by our group roadmaps our imaging approach in the evaluation of such patients.^9^ In accordance with our proposed protocol, recent data also favor medical pretreatment with pulmonary vasodilators prior to BPA therapy to reduce complications.^11^

The intraprocedural phase of our protocol focuses on preferential lesion selection with appropriate balloon sizing, prioritization of segmental interventions, and judicious contrast administration. Subselective segmental angiography with visual balloon undersizing, to avoid vascular injury, remains the standard with adjunctive pressure gradient assessment especially for hard to image intravascular web-like lesions.^14^^,^^21^ Balloon dilation until brisk venous return is achieved remains the gold standard for adequate dilation.^14^^,^^15^ In comparison with other registries, where up to 10 segmental arteries are frequently treated per session, we favor a refined approach.^22^ Limiting interventions to 3 to 4 segments or less per session helps to minimize the reperfusion pulmonary edema risk and facilitates safe outpatient postprocedural observation.^16^^,^^22^ Lastly, judicious hand injections of diluted contrast helps minimize contrast dose during procedure sessions.^12^

The postprocedural phase emphasizes early identification of complications with necessary contingencies on standby. Patients are observed in an outpatient telemetry capable bed. After 8 to 23 hours of uncomplicated observation, the patient is able to return home. After 2 sequential BPA sessions, all patients receive routine follow-up in a virtual setting to closely assess for complications.

Our protocol was borne out of necessity because of the health care constraints of the COVID-19 pandemic. The protocol also follows a precedent set by other interventional procedures, including transcatheter aortic valve replacement and percutaneous coronary intervention, which have safely transitioned to an outpatient model.^8^^,^^23^ With continued familiarity and experience we predict BPA will likely transition entirely to an outpatient protocol, including same day discharge at experienced centers.

### Limitations

Because this study was conducted retrospectively on a limited number of patients at a single center, our data should be confirmed with a larger, multicenter, prospective study. In addition, standardization of medical therapy, including pulmonary vasodilator therapy, was not uniform across all patients.

### Future directions

Access to medical care can be conceptualized through the 5 A’s: approachability, acceptability, availability, affordability, and appropriateness.^24^ Although BPA is gaining recognition and therapeutic evidence as a viable option for patients with CTEPH, availability is becoming one of the greatest limiting factors. The next frontier of BPA therapy is continued dissemination of knowledge regarding technical expertise to allow ease of access, similar to the advent of other interventional procedures.

## Conclusion

BPA can be effectively and safely performed in the outpatient setting with a standardized protocol that has the necessary contingencies readily available.
